# Two Glycosyltransferase Genes of *Haemophilus parasuis* SC096 Implicated in Lipooligosaccharide Biosynthesis, Serum Resistance, Adherence, and Invasion

**DOI:** 10.3389/fcimb.2016.00100

**Published:** 2016-09-12

**Authors:** Qi Zhou, Saixiang Feng, Jianmin Zhang, Aiqing Jia, Kaijie Yang, Kaixiang Xing, Ming Liao, Huiying Fan

**Affiliations:** ^1^Key Laboratory of Veterinary Vaccine Innovation of the Ministry of Agriculture, College of Veterinary Medicine, South China Agricultural UniversityGuangzhou, China; ^2^Guangdong Haid Institute of Animal Husbandry and VeterinaryGuangzhou, China

**Keywords:** *Haemophilus parasuis*, lipooligosaccharide, glycosyltransferase, serum resistance, adhesion and invasion

## Abstract

*Haemophilus parasuis* is a common opportunistic pathogen known for its ability to colonize healthy piglets and causes Glässer's disease. The lipooligosaccharide (LOS) of *H. parasuis* is a potential virulence-associated factor. In this study, two putative glycosyltransferases that might be involved in LOS synthesis in *H. parasuis* SC096 were identified (*lgtB* and *lex-1*). Mutants were constructed to investigate the roles of the *lgtB* and *lex-1* genes. The LOS from the Δ*lgtB* or Δ*lex-1* mutant showed truncated structure on silver-stained SDS-PAGE gel compared to the wild-type strain. The Δ*lgtB* and Δ*lex-1* mutants were significantly more sensitive to 50% porcine serum, displaying 15.0 and 54.46% survival rates, respectively. Complementation of the *lex-1* mutant restored the serum-resistant phenotype. Additionally, the Δ*lgtB* and Δ*lex-1* strains showed impaired ability to adhere to and invade porcine kidney epithelial cells (PK-15). The above results suggested that the *lgtB* and *lex-1* genes of the *H. parasuis* SC096 strain participated in LOS synthesis and were involved in serum resistance, adhesion and invasion.

## Introduction

*Haemophilus parasuis* is an important porcine pathogen and the etiological agent of Glässer's disease, which is characterized by fibrinous polyserositis, polyarthritis, and meningitis. It is a commensal organism found in the upper respiratory tract of swine that causes systemic symptoms in conditions with decreased resistance (Oliveira and Pijoan, 2004). The exact mechanisms by which *H. parasuis* invades internal organs to cause local and disseminated infection are not fully understood.

Lipooligosaccharide (LOS) has been identified as a potential *H. parasuis* virulence factor, however, only one investigation has analyzed the role of antigenic structure of the *H. parasuis* LOS (Xu et al., 2013). Most LOS molecules consist of two main components: lipid A and a nonrepeating core oligosaccharide. The core oligosaccharide components are typically 3-deoxy-D-manno-octulosonic acid (Kdo), heptose (Hep), glucose (Glu), galactose (Gal), and phosphate. The backbone of the lipid A moiety is substituted at position 6′ with a 2,4-linked Kdo disaccharide, which serves as an acceptor for the transfer of the first heptose residue to position 5 of the first Kdo residue; this transfer is accomplished by the heptosyltransferase family (Gronow et al., 2005). A lack of genes encoding heptosyltransferases often prevents the incorporation of the heptose residue and subsequently blocks the addition of other sugar moieties, resulting in truncated LOS in bacteria, including *Haemophilus influenzae, Haemophilus ducreyi*, and *Campylobacter jejuni* (Gibson et al., 1997; Gronow et al., 2005; Naito et al., 2010). In *H. parasuis*, deletion of the *opsX, rfaF*, and *waaQ* genes, which encode the three heptosyltransferases, produced severely truncated LOS structures, decreased resistance to complement-mediated killing in serum and a decreased ability to adhere to and invade porcine kidney epithelial (PK-15) and porcine umbilical vein-derived endothelial cells (PUVECs) (Xu et al., 2013). However, other glycosyltransferases associated with LOS biosynthesis and pathogenesis have yet to be investigated.

Glycosyltransferase family 25 (NCBI accession no. cd06532) has been reported to be involved in LOS biosynthesis (Jennings et al., 1995; Edwards et al., 2005; Masoud et al., 2008). Here, two putative glycosyltransferase family 25 genes (*lgtB* and *lex-1*) were identified in *H. parasuis* SC096 by sequencing analysis. The *lgtB* genes from *Neisseria meningitidis* and *Neisseria gonorrhoeae* encode the β-1,4-galactosyltransferase required for LOS core biosynthesis and show homology to the galactosyltransferases from *Pasteurella haemolytica, H. ducreyi, Haemophilus sommnus* (GenBank accession no. AF096997), and *H. influenzae* (High et al., 1993; Potter and Lo, 1995; Sun et al., 2000; Park et al., 2002). In *H. influenzae* type B, the *lex-1* gene is involved in LOS biosynthesis and virulence. Genetic transformation using the cloned *H*. *influenzae* type b DNA fragment containing *lex-1* increased the virulence in virulence-deficient LOS mutants (Cope et al., 1991; Ma et al., 1996). However, whether the *lgtB* or *lex-1* gene of *H. parasuis* participates in LOS biosynthesis and disease pathogenesis is unknown. In this study, we generated Δ*lgtB* and Δ*lex-1* mutants of the *H. parasuis* SC096 strain to investigate their roles in serum resistance, host cell adherence, and invasion.

## Materials and methods

### Bacterial strains, plasmids, and growth conditions

The bacterial strains and plasmids used in this study are described in Table 1. *Escherichia coli* plasmids were propagated in *E. coli* DH5α grown in Luria-Bertani medium (Oxoid) at 37°C. *H. parasuis* clinical strain SC096 was cultured on Trypticase Soy Agar (TSA) or Trypticase Soy Broth (TSB) (Oxiod) supplemented with 0.002% (w/v) nicotinamide adenine dinucleotide (NAD; Sigma) and 5% (v/v) inactivated bovine serum at 37°C in a 5% CO_2_-enriched atmosphere. For selection of the plasmid-containing strains, the medium were supplemented with 30 μg/mL of kanamycin or gentamycin.

**Table 1:** **Bacterial strains and plasmids used in this study**.

**Strain or plasmid**	**Relevant characteristic(s)**	**Source**
**STRAINS**		
*E. coli* DH5α	F^−^, ϕ80d/*lacZ*ΔM15, Δ(*lacZYA*-*argF*) U169 *recA*1 *endA*1 *hsdR*17	Laboratory collection
*H. parasuis* SC096	Serovar 4 clinical isolate	Zhang et al., 2012b
Δ*lgtB*	SC096 Δ*lgtB*::Kan^R^	This study
Δ*lex-1*	SC096 Δ*lex-1*::Kan^R^	This study
Δ*lgtB*-c	SC096 complemented Δ*lgtB* strain, Gm^R^ Kan^R^	This study
Δ*lex-1*-c	SC096 complemented Δ*lex-1* strain, Gm^R^ Kan^R^	This study
Δ*lgtB*-np	SC096 Δ*lgtB*::Gm^R^, in-frame non polar deletion	This study
Δ*lgtB*-oc	SC096 complemented Δ*lgtB* strain, Gm^R^ Kan^R^, original locus complement	This study
**PLASMIDS**		
pMD-19T (simple)	T-vector, AmpR	Takara Inc.
pK18mobsacB	Suicide and narrow-broad-host vector, Kan^R^	Schäfer et al., 1994
pSF115	Kan resistance cassette-carrying complement vector, Kan^R^	Zou et al., 2013
p34S-Gm	Gm resistance cassette-carrying vector, Gm^R^	Laboratory collection
pSF116	Gm resistance cassette-carrying complement vector, Gm^R^	This study
pZQ001	A 1937bp fragment containing Kan^R^, the upstream and downstream sequences of the *lgtB* gene in pMD 19T(simple), Kan^R^	This study
pZQ002	A 2076bp fragment containing Kan^R^, the upstream and downstream sequences of the *lex-1* gene in pMD 19T(simple), Kan^R^	This study
pZQ003	A 1398bp fragment containing Gm^R^ and the *lgtB* gene in pSF116	This study
pZQ004	A 1460bp fragment containing Gm^R^ and the *lex-1* gene in pSF116	This study
pZQ005	A 1786bp fragment containing Gm^R^, the upstream and downstream sequences of the *lgtB* gene in pMD 19T(simple), Gm^R^	This study
pZQ006	A 2624 bp fragment containing Gm^R^ and the *lgtB* gene in pMD 19T(simple), Gm^R^	This study

### Construction and complementation of the *lgtB* and *lex-1* mutants

The oligonucleotides used for PCR are listed in Table 2. A DNA fragment encompassing the upstream region of the *lgtB* gene was amplified using the primer pair P1 and P2. The region downstream of the *lgtB* gene was amplified using the primer pair P3 and P4. A kanamycin resistance (Kan^R^) cassette was amplified from pK18mobsacB using primers P9 and P10. These three fragments were connected by overlap PCR with primers P1 and P4 and then ligated into pMD-19T (simple) to obtain the plasmid pZQ001. Natural transformation was used to introduce pZQ001 into SC096 to obtain the *lgtB* mutant following a previously described method (Zhang et al., 2012a). The *lex-1* mutant was constructed in the same manner with different primers. Primers P5 and P6 were used to amplify the upstream region of *lex-1*, primers P7 and P8 were used to amplify the downstream region, and primers P9 and P10 were used to amplify the Kan^R^ cassette. The three fragments were amplified using primers P5 and P8 by overlap PCR and then ligated into pMD-19T (simple) to obtain the plasmid pZQ002. Finally, the plasmid was introduced into SC096 using natural transformation then generated the *lex-1* mutant. The mutants were confirmed by PCR and sequencing.

**Table 2:** **Sequences of the PCR primers used in this study**.

**Primers**	**Primer sequences(5′-3′)**
P1 (*lgtB* up-F)	ATACCGCTTGTGTGTGAGCGTCTTATATCAGCT
P2 (*lgtB* up-R)	ATGTCAATTCGGGATCCGCGTCTACTTCAGTAAGCGAA
P3 (*lgtB* down-F)	GATCGGCTTCGTCGACACGTTCGTATGTAGGAGCTGCTGGAT
P4 (*lgtB* down-R)	AGGGTAGAAGCACTCATATAG
P5 (*lex-1* up-F)	ATACCGCTTGTGTCACCTAAGATAATATCATC
P6 (*lex-1* up-R)	ATGTCAATTCGGGATCCGCGTATGTGAGCGTCTTATATCAG
P7 (*lex-1* down-F)	GATCGGCTTCGTCGACACGTTCGCTCCTATTAATGGTAG
P8 (*lex-1* down-R)	GTAGCTCAGAATGATTATCGCCA
P9 (Kan-F)	CGCGGATCCCGAATTGACAT TTTTATGGACAGCAAGCGAA
P10 (Kan-R)	ACGTGTCGACGAAGCCGATC TCAGAAGAACTCGTCAAGAA
P11 (*lgtB* comp-F)	GGTTCAAAAGAAGTTTCTATGTAAGAGTTAATTCATATTGAAGG
P12 (*lgtB* comp-R)	ATGTCAATTCGGGATCCCTATTTAAATTCAACAGTTC
P13 (*lex-1* comp-F)	GGTTCAAAAGAAGTTTCTATGTAATATGCTATCTTAGCATAAAG
P14 (*lex-1* comp-R)	ATGTCAATTCGGGATCC TTATTCAAAAGGAATAATAC
P15 (Gm^R^-R)	GCGGTACTTGGGTCGATATC
P16 (Gm^R^ BamHI-F)	CGCGGATCCCGAATTGACATCGAATTGACATAAGCCTGTTC
P17 (Gm^R^ SalI-R)	ACGTGTCGACGAAGCCGATCTTAGGTGGCGGTACTTGGGTC
**Expression studies by quantitative RT-PCR**	
P18 (*lgtB*-F)	GACTGGTTTGAGCATTTAGATG
P19 (*lgtB*-R)	TCTAATACAGAATAGCGGG
P20 (*rplM*-F)	GTGACTGGTATGTAGTAG
P21 (*rplM*-R)	TGCCACCTACATAGCCAG
P22 (*lgtB*-F for test)	AATATCTTCTGCTTCCAAGG
P23 (*lgtB*-R for test)	CAATCAATCGGTGTTTTCTG
P24 (*lgtB*-up-F for non polar deletion)	ACCGCTTGTGTGCCGTACCATAATGTTTAG
P25 (*lgtB*-up-R for non polar deletion)	TATAATTTCCTTCAATATGAAT
P26 (*lgtB*-down-F for non polar deletion)	TGAAAAATATTACATATGTATTTG
P27 (*lgtB*-down-R for non polar deletion)	AATTGCGTTGCAGTACAAGC
P28 (Gm^R^-F for non polar deletion)	ATTCATATTGAAGGAAATTATAATGTTACGCAGCAGCAACGA
P29 (Gm^R^-R for non polar deletion)	CAAATACATATGTAATATTTTTCATTAGGTGGCGGTACTTGGGTC
P30 (*lgtB*-up-R for original locus complementation)	CTATTTAAATTCAACAGTTCT
P31 (Gm^R^-F for original locus complementation)	AGAACTGTTGAATTTAAATAGCGAATTGACATAAGCCTGTTC

The pSF116 vector was constructed as follows. A 554-bp DNA fragment including the gentamicin resistance (Gm^R^) gene was amplified from p34S-Gm using the primer pair P16 and P17. Then the Gm^R^ gene and pSF115 were digested with BamHI and SalI. The two digested products were ligated to obtain the complement vector pSF116. To construct the complementing plasmids pZQ003 and pZQ004, the *lgtB* and *lex-1* genes were amplified from SC096, then cloned into KpnI and BamHI-digested pSF116 using the In-fusion HD cloning kit (Clontech Laboratories, TaKaRa Bio Inc., Shiga, Japan) respectively.

To construct an in-frame non-polar *lgtB* mutant, A DNA fragment about 500-bp encompassing the upstream region of the *lgtB* was amplified using the primer pair P24 and P25, whereas the downstream DNA region of the *lgtB* was amplified using the primer pair P26 and P27. An intact gentamicin resistant (Gm^R^) cassette (from ATG to TAA) was amplified by PCR from p34S-Gm using primers P28 and P29. These three fragments were connected by overlap PCR with the primers P24 and P27, then purified and ligated into pMD19-T to give plasmid pZQ005. Then the plasmid was introduced into SC096 using natural transformation to construct a mutant containing a non-polar, in frame mutation in *lgtB*, Δ*lgtB*-np. This mutant was confirmed by PCR and sequenced using primer P22 and P23.

To construct a strain in which the wild-type *lgtB* gene is restored in the *lgtB*::kan mutant, the fragment containing upstream region of the *lgtB* and the intact *lgtB* was amplified using the primer pair P24 and P30. The downstream region fragment was amplified using the primer pair P26 and P27. The gentamicin resistant (Gm^R^) cassette was amplified by PCR from p34S-Gm using primers P31 and P29. The three fragments were connected using primers P24 and P27 by overlap PCR and then ligated into pMD-19T (simple) to obtain the plasmid pZQ006. The plasmid was transformed into *lgtB* insertion mutant (Δ*lgtB*::Kan^R^) to get the *lgtB* original locus complement strain. This complement strain was confirmed by PCR and sequenced using primer P22 and P23.

### Growth studies

To obtain growth curves for the wild-type SC096 strain, *lgtB* or *lex-1* mutant, cultures of each strain were grown overnight in TSB supplemented with NAD and 5% serum. Then the cultures were inoculated into fresh TSB medium supplemented with NAD and 5% serum at a ratio of 1: 100 and incubated at 37°C. The optical density at 600 (OD600) was measured at 1 h intervals.

### LOS preparation

Extraction of LOS with high purity was performed using a modified phenol-water extraction protocol accompanied by proteinase K digestion of the bacterial proteins and nuclease elimination of the nucleic acids (Hitchcock and Brown, 1983). The LOS preparations were treated with sodium dodecyl sulfate (SDS) loading buffer (100 mM Tris–HCl, pH 8.0, 2% β-mercaptoethanol, 4% SDS, 0.2% bromophenol blue, 0.2% xylene cyanole, and 20% glycerol), separated by SDS-polyacrylamide gel electrophoresis (PAGE; 4% stacking gel and 15% separating gel) at 100 V for 1 h and visualized by silver staining.

### Serum bactericidal assays

Porcine serum was collected from four healthy piglets (3–4 weeks old) from a farm free of Glässer's disease and was stored at −80°C. Some serum aliquots were treated at 56°C for 30 min to inactivate complement. The serum bactericidal assay was performed as described by (Zhang et al., 2012a). Briefly, bacterial suspensions [10^7^–10^8^ colony-forming units (CFU)/mL] were cultured with either fresh porcine serum or heat-inactivated serum at 1:1 ratios or different serum concentrations for 1 h. After incubation, 10-fold serial dilutions of the samples were generated, spotted onto plates and incubated at 37°C for 48 h. Then, the bacterial numbers were counted, and the survival ratios were calculated. The results were expressed as the means of triplicates from three independent experiments.

### Adhesion and invasion assays

PK-15 cells were used for the adhesion and invasion assays following the previously described method (Xu et al., 2013). The cells (5 × 10^5^ CFU/mL) were seeded into 24-well tissue culture plates in Dulbecco's modified Eagle's medium (DMEM; Invitrogen) containing 10% heat-inactivated fetal bovine serum. The cells were cultured at 37°C in a humidified incubator with 5% CO_2_ for 24 h, washed twice with PBS and then infected with approximately 1 × 10^7^ CFU of *H. parasuis*. The culture plates were incubated for up to 2 h at 37°C to allow bacterial adhesion. The cells were rigorously washed five times with PBS to eliminate non-specific bacterial attachment and were then incubated for 10 min at 37°C with 100 mL of 0.25% trypsin/EDTA. After incubation, 900 μL of ice-cold TSB was added, the cells were removed from the culture plates by scraping the bottoms of the wells. Bacterial enumeration was performed using serial 10-fold dilutions and plating on TSA plates. For the invasion assay, cell culture, bacterial infection, and bacterial counting were performed as described above for the bacterial adherence assay except that the extracellular bacteria were killed by incubation of the monolayer with DMEM containing chloromycetin (25 μg/mL) for another 2 h following the incubation with the bacteria and three washes with PBS. All of the above assays were performed in triplicate and repeated three times.

### Quantitative real-time PCR

RNAs were isolated from the SC096 and Δ*lgtB*-c strain. The *lgtB* gene transcripts were analyzed by quantitative reverse transcription PCR (qRT-PCR). RNA was extracted using the bacterial RNA kit (Omega, USA) according to the manufacturer's instructions. The reactions were performed with the one-step SYBR® PrimeScript™ PLUS RT-PCR Kit (Clontech, USA). The 2(−ΔΔC(T)) method was used to relatively quantitate *lgtB* gene expression compared to the stably expressed *rplM* reference gene. The 7500 Real-Time PCR System (Applied Biosystems, Carlsbad, CA, USA) was used for the assay.

### Statistical analysis

All experiments were repeated three times, and the results were expressed as means ± the standard deviations (*SD*). To determine significance of obtained results, comparison between groups was made using Student *t*-test. *P* < 0.01 were considered statistically significant.

## Results

### Analysis of the *lgtB* and *lex-1* gene sequences

Two putative lipooligosaccharide biosynthesis genes (*lgtB* and *lex-1*) from *H. parasuis* SC096 were sequenced and identified using the BLAST program. The *lgtB* gene had 789 bp and showed 100% identity with a glycosyltransferase from *H. parasuis* ZJ0906 (NCBI accession no. AGO15727), whereas *lex-1* had 837 bp and showed 96% identity with another glycosyltransferase from *H. parasuis* ZJ0906 (NCBI accession no. AGO15728).

**Figure 1:**
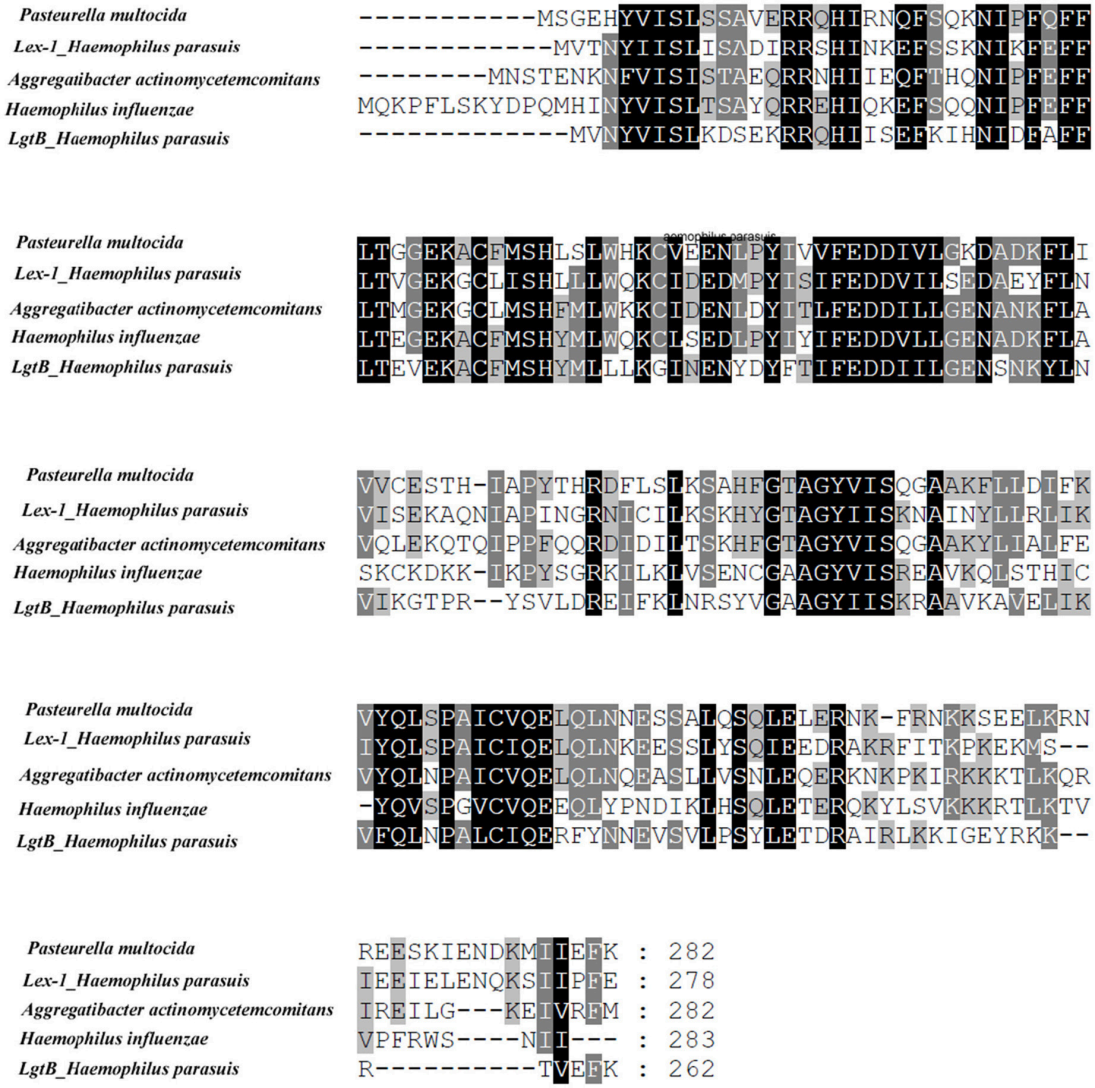
**Multiple alignments of the ***lex-1*** or ***lgtB*** amino acid sequences from ***H. parasuis*** SC096 with those from ***Pasteurella multocida*** (no. WP_014390682.1), ***Aggregatibacter actinomycetemcomitans*** (no. WP_053330004.1), ***Haemophilus influenza*** (no. WP_048952714.1)**. Shadowed letters indicated either identical residues or conservative changes.

A global sequence comparison between the known *lgtB* and *lex-1* proteins indicated that they belonged to glycosyltransferase family 25. *lgtB* showed 41, 42, and 41% identities with the β-1,4 galactosyltransferase from *Pasteurella multocida* (no. WP_014390682.1), the lipooligosaccharide biosynthesis protein *lex-1* from *Aggregatibacter actinomycetemcomitans* (no. WP_053330004.1) and the lipooligosaccharide biosynthesis protein *lic2B* from *H. influenzae* (no. WP_048952714.1), respectively. *lex*-1 showed 45, 46, and 46% identities with the same sequences, respectively. Multiple alignments were created using CLUSTAL W (Figure 1).

### Construction of the *H. parasuis* Δ*lgtB*/Δ*lex-1* mutants and complemented strains

**Figure 2:**
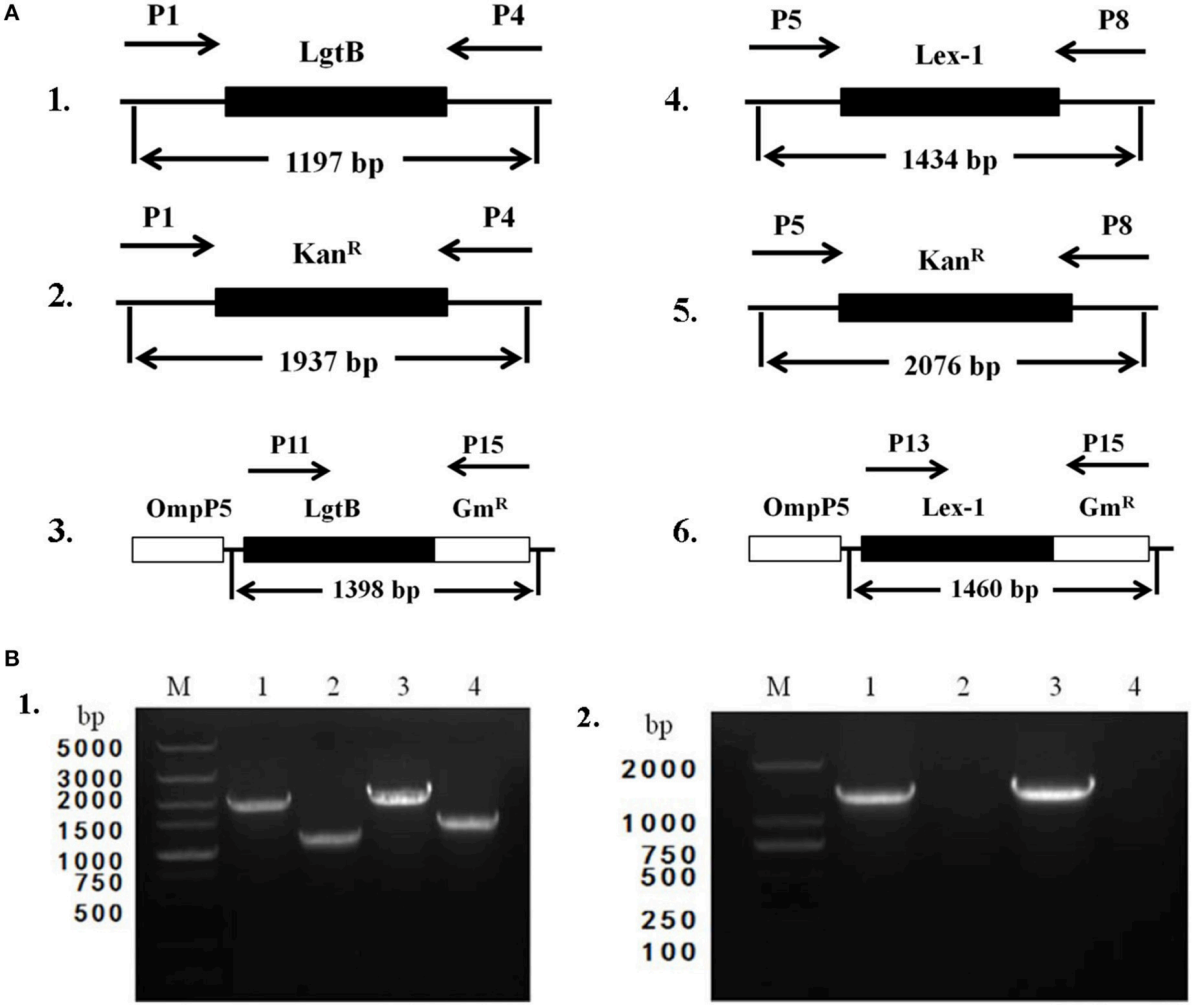
**Construction and characterization of Δ***lgtB*** and Δ***lex-1*** mutants and complemented strains. (A)** Parts 1–3 show the maps of the *lgtB* gene of wild-type SC096, the *lgtB*::Kan^R^ insertion mutant and its complemented strain. Parts 4–6 show the maps of the *lex-1* gene of wild-type SC096, the *lex-1*::Kan^R^ insertion mutant and its complemented strain. **(B)** Part 1, Primers P1 and P4 were used to amplify upstream to downstream of *lgtB* from the Δ*lgtB* mutant strain (lane 1), the wild type SC096 (lane 2); Primers P5 and P8 were used to amplify upstream to downstream of *lex-1* from the Δ*lex-1* mutant strain (lane 3), the wild type SC096 (lane 4). Part 2, Primers P11 and P15 were used to amplify *lgtB* and Gm^R^ from the Δ*lgtB*-c strain (lane 1), the wild type SC096 (lane 2); Primers P13 and P15 were used to amplify *lex-1* and Gm^R^ from the Δ*lex-1*-c strain (lane 3), the wild type SC096 (lane 4).

To obtain the Δ*lgtB* mutant, pZQ001, which contained the Δ*lgtB*::Kan^R^ insertion, was introduced into the SC096 strain by natural transformation. To obtain the Δ*lex-1* mutant, pZQ002, which contained the Δ*lex-1*::Kan^R^ insertion, was introduced in the same manner. To determine the phenotypes of the *lgtB* or *lex-1* strains due to the inactivation of the *lgtB* and *lex-1* genes, pZQ003 was introduced into the Δ*lgtB* mutant by transformation to obtain a Δ*lgtB*-Comp strain, and pZQ004 was introduced to obtain a Δ*lex-1*-Comp strain. In the complemented strains, the intact *lgtB* or *lex-1* with a gentamycin resistance cassette was inserted immediately downstream of *ompP5*. Analysis of the transformants indicated that the *lgtB* and *lex-1* genes plus a kanamycin resistance cassette were integrated into the homologous chromosomes of the mutant strains (Figure 2A). Colony PCR was used to confirm the transformants (Figure 2B). The ability of these mutants to grow under standard conditions was tested, but only negligible changes in the growth rates were detected (Figure S1).

### Denaturing gel electrophoresis of the lipooligosaccharides

**Figure 3:**
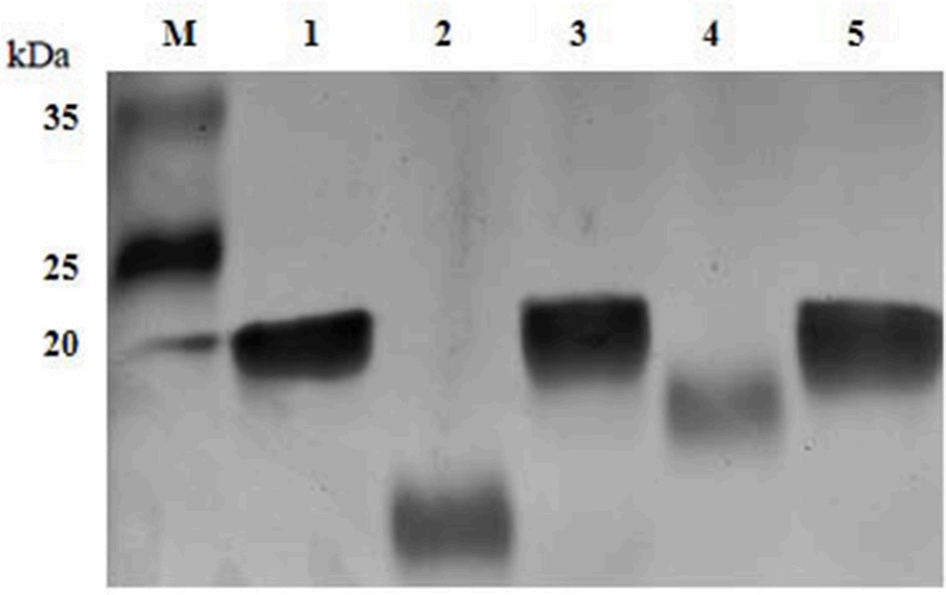
**LOS profiles of ***H. parasuis*** SC096 strain, mutants, and complemented strains**. Lane 1, wild-type SC096; Lane 2 and 3, Δ*lgtB* and its complemented strain Δ*lgtB*-c; Lane 4 and 5, Δ*lex-1* and its complemented strain Δ*lex-1*-c.

To evaluate the variations in the LOS glycoforms, the LOSs of the wild-type SC096 strain, mutants and complemented strains were assessed by SDS-PAGE. Electrophoretic analysis of the LOS from the Δ*lgtB* or Δ*lex-1* mutant indicated that the profiles had changed compared to the profile of the parental strain SC096 (Figure 3). The SC096 strain had a LOS band at 20 kDa (Lane 1), whereas the Δ*lgtB* mutant had a LOS with a distinctly smaller molecular mass (Lane 2). Specifically, the LOS of the Δ*lgtB* or Δ*lex-1* mutant (Lanes 2 and 4) migrated faster than the LOS from the wild-type SC096 strain, and the LOS from the Δ*lgtB* mutant migrated even faster than the LOS from the Δ*lex-1* mutant. The LOSs of the complemented strains (Lanes 3 and 5) were similar or identical to the LOS of the parent strain, which suggested that LOS synthesis was restored in the complemented strains. These results suggested that *lgtB* and *lex-1* might affect the LOS structures.

### Resistance to complement-mediated serum killing

**Figure 4:**
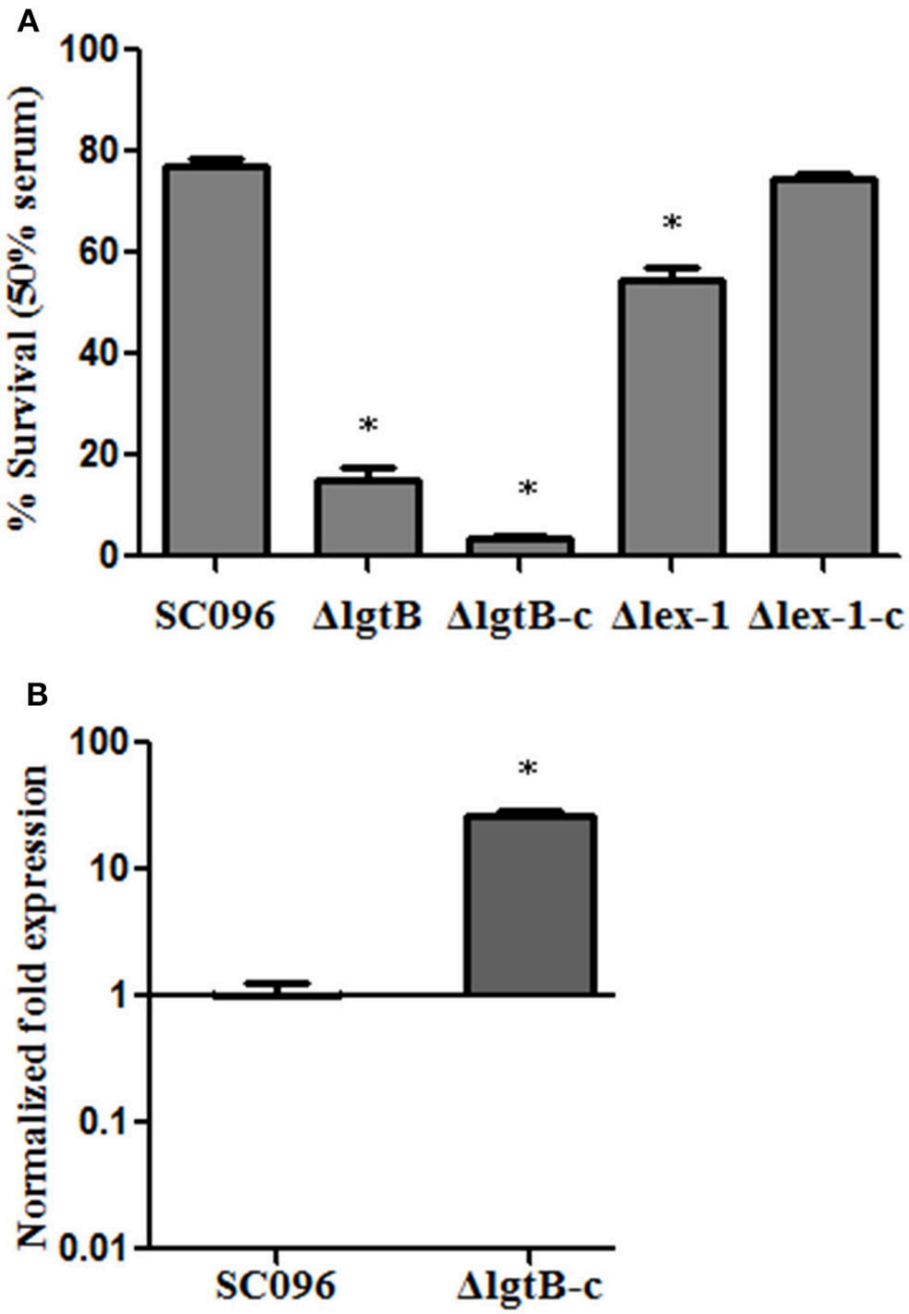
**Survival of ***H. parasuis*** strains in 50% porcine sera (A)**. The survival percentage was calculated as the ratio of colonies in fresh serum to those in heat-treated serum. Error bars represent the standard deviation from three independent experiments. The asterisks indicate that the survival of bacteria in serum was statistically different (*p* < 0.01) from that of the wild-type SC096 strain as judged by the Student *t*-test. qRT-PCR analysis of mRNA levels of *lgtB* in Δ*lgtB*-c mutant compared with mRNA levels wild-type SC096 **(B)**. The data represent means standard errors (*n* = 3). The asterisks indicate that mRNA levels of *lgtB* in the complemented strain was statistically different (*p* < 0.01) from that of the wild-type strain as judged by the Student *t*-test.

**Figure 5:**
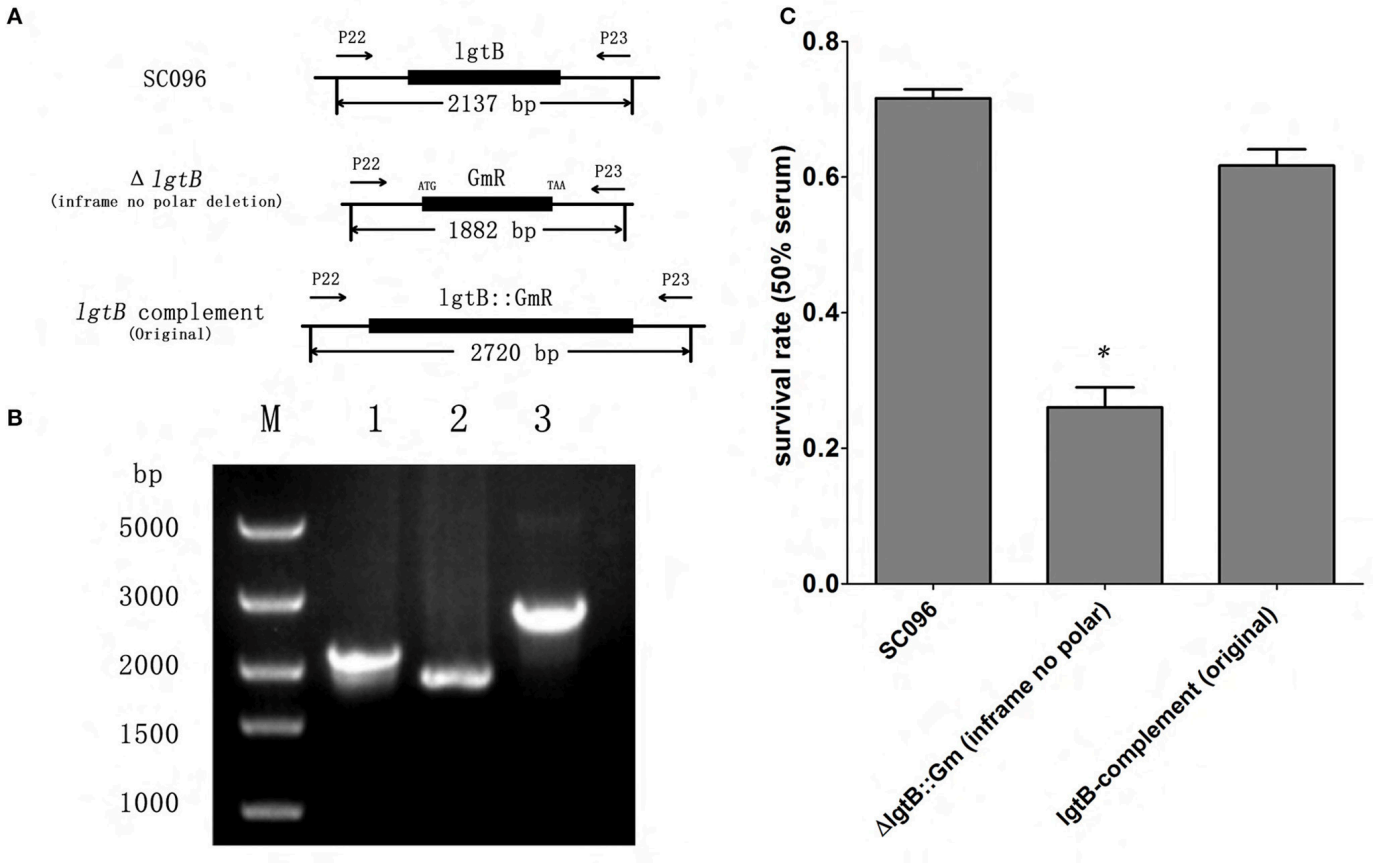
**Construction and verification of ***lgtB*** in-frame non polar mutant and the original complemented strain. (A)** Schematic of *lgtB* in-frame non polar mutant and the original complemented strain. The *lgtB* in-frame non polar mutant was constructed by replacing *lgtB* with Gm^R^ gene from ATG to TAA codon. The original complemented strain was constructed by inserting the intact *lgtB* gene follow by Gm^R^ gene into previous constructed Δ*lgtB*::Kan^R^ strain in order to confirm the phenotype of the previous *lgtB*::Kan^R^ mutant. **(B)** PCR analysis verifying the *lgtB* in-frame non polar mutant and the original complemented strain. Primers P22 and P23 were used to amplify the locus region of *lgtB* from the wild type SC096 (lane 1), the *lgtB* in-frame non polar strain (lane 2), and the original complemented strain (lane 3); lane M shows a 5Kb DNA molecular marker. **(C)** Survival of *lgtB* in-frame non polar mutant and the original complemented strains treated with 50% porcine serum. The *lgtB* in-frame non polar mutant showed significantly increased susceptibility to serum compared with the wild type strain SC096 (*p* < 0.01) with 50% porcine serum, while the original complemented strain restored the serum resistant phenotype. Error bars represent the standard deviation of three independent experiments. The asterisks indicate that the survival of bacteria in serum was statistically different (*p* < 0.01) from that of the wild-type SC096 strain as judged by the Student *t*-test.

To investigate whether the *lgtB* and *lex-1* genes were involved in serum resistance, the survival rates of Δ*lgtB*, Δ*lex-1*, and their complemented strains were assessed in 50% porcine serum (Figure 4A). Compared to the wild-type SC096 strain, the Δ*lgtB* and Δ*lex-1* mutants were both significantly more sensitive to pig serum (*p* < 0.01), resulting in survival rates of 15.0 and 54.46% in 50% pig serum, respectively. The Δ*lgtB* mutant was more susceptible to pig serum than the Δ*lex-1* strain (*p* < 0.01). Furthermore, *lex-1* mutant showed significantly increased susceptibility to serum of different concentrations compared with the wild type strain SC096 (*p* < 0.01; Figure S2). Interestingly, complementation of the *lex-1* mutant restored the serum resistance phenotype, whereas the survival rate following complementation of the *lgtB* mutant was 8.51%, representing an approximately 10-fold reduction in 50% porcine serum susceptibility. A qRT-PCR analysis was performed to confirm the transcription level of the *lgtB* gene in the complemented strain (Figure 4B). The Δ*lgtB*-c strain exhibited *lgtB* transcription level that was increased 27.05-fold relative to the SC096 strain, indicating that *lgtB* was overexpressed in the complemented strain. In order to confirm the phenotype of *lgtB* mutant, a new in-frame deletion non-polar mutant (Δ*lgtB*::Gm^R^) and an original locus complemented strain were constructed (Figures 5A,B). The new mutant was as sensitive to pig serum as the previous, whereas the original locus complemented strain restored the serum resistance phenotype (Figure 5C). The results suggested that *lgtB* and *lex-1* may be associated with serum resistance.

### Adherence and invasion abilities

**Figure 6:**
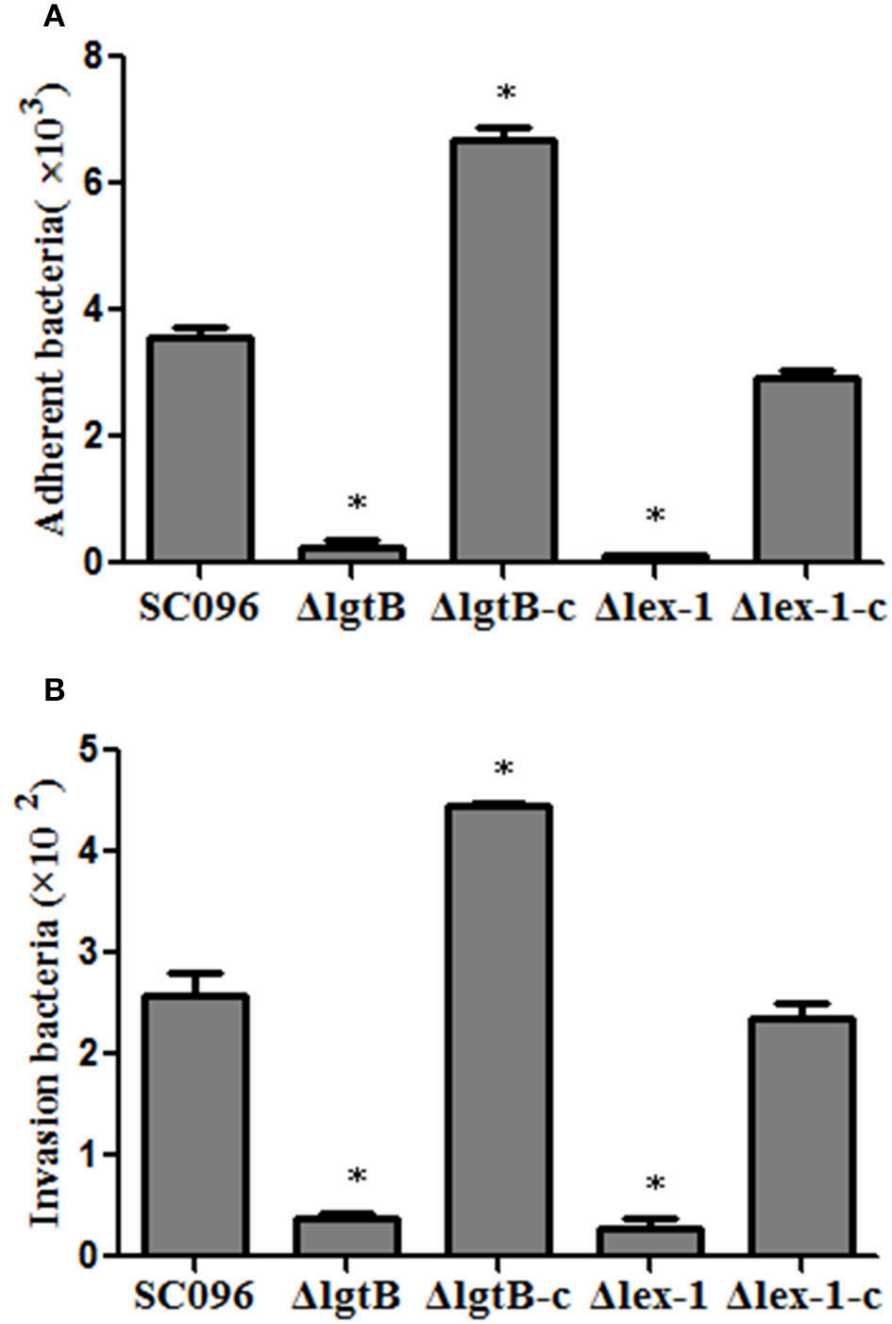
**Adherence (A) and invasion (B) of ***H. parasuis*** wild-type SC096, Δ***lgtB*** and Δ***lex-1*** mutants and complemented strains in PK-15 cells**. The data represented the number of bacteria that adhered to or invaded the cells in each well of a 24-well plate. Error bars represent the standard deviation from three independent experiments performed in triplicate. The asterisks indicate that the number of bacteria bound to the PK-15 cells was statistically different (*p* < 0.01) from that of the wild-type SC096 strain as judged by the Student *t*-test.

To assess the effects of the *H. parasuis lgtB* and *lex-1* genes on host cell interactions, PK-15 cells were incubated with the wild-type, mutant, and complemented strains to compare the adherence and invasion abilities. As illustrated in Figure 6A, there was significantly less adhesion by the Δ*lgtB* and Δ*lex-1* mutants than the wild-type SC096 strain (*p* < 0.01). Similarly, the Δ*lgtB* and Δ*lex-1* mutants showed significantly less invasion efficiency (Figure 6B; *p* < 0.01). The adhesion and invasion levels were fully recovered in the complemented *lex-1* strain. The complemented *lgtB* strain exhibited a 1.87-fold increase in adherence and a 1.64-fold increase in invasion. The results indicated that *lgtB* and *lex-1* have an effect on the ability of the bacteria to interact with the host cells.

## Discussion

Previous investigations have shown that the *H. parasuis* LOS has a significant influence on pathogenesis, including pathogen adherence, invasion, serum resistance, and endotoxicity (Amano et al., 1997; Tadjine et al., 2004; Zhang et al., 2014). Analysis of LOS biosynthesis and structures could contribute to explorations of the relationship between the LOS components and pathogenesis (Perry et al., 2013). One study presented evidence that the heptosyltransferases that transfer the heptose I and heptose II residues contributed to the virulence-associated properties of *H. parasuis* (Xu et al., 2013). Additionally, some galactosyltransferases are involved in the biosynthesis of LOS and virulence in certain pathogenic bacteria. For instance, β-1,4-galactosyltransferases encoded by the *lgtB* gene of *N. meningitidis* are required for the addition of least three sugars in the lacto-N-neotetraose chain (Park et al., 2002). In *H. influenzae*, the *lex2* locus is identified as a phase-variable LPS biosynthetic locus and contributes to resistance of the bacteria to the killing effect of serum (Griffin et al., 2005; Deadman et al., 2009). Moreover, *lic2B* gene, which is required for addition of a galactose residue to the LOS outer core, is crucial for optimal survival of nontyeable *H. influenzae* in a mouse model of bacteremia and for evasion of serum complement (Wong et al., 2011). In *H. ducreyi*, the *lbgAB* genes are involved in lipooligosaccharide biosynthesis and serve as one indicator of the classification of *H. ducreyi* strains (Stevens et al., 1997; Tullius et al., 2002). To examine whether the *lgtB* and *lex-1* of *H. parasuis* genes participate in LOS biosynthesis, we constructed chromosomal knockout Δ*lgtB* and Δ*lex-1* mutants of the *H. parasuis* SC096 strain. Deletion of either the *lgtB* or the *lex-1* gene caused a truncated LOS profile on silver-stained SDS-PAGE gel, which demonstrated that *lgtB* and *lex-1* were necessary for lipooligosaccharide biosynthesis in the SC096 strain. The phenotypic differences in the LOS patterns following the deletion of the *lgtB* and *lex-1* genes in the SC096 strain could be attributed to the substrate specificity of the two glycosyltransferases.

Serum resistance is an important bacterial pathogenic mechanism. In *H. influenzae*, both lipooligosaccharide and capsular polysaccharide contribute to resistance against complement-mediated attacks and, hence, the increased survival of *H. influenzae* (Hallström and Riesbeck, 2010). In *H. parasuis*, LOS and the polysaccharide biosynthesis protein CapD have been reported to participate in resistance to complement-dependent bactericidal activity (Wang et al., 2013; Xu et al., 2013). Loss of heptose I or heptose II from LOS resulted in notable defects in serum resistance, and deletion of CapD significantly attenuated the serum resistance ability and pathogenicity of *H. parasuis*. Here, sensitivity to complement following the loss of the *lgtB* gene in the SC096 strain indicated that *lgtB* gene expression was associated with serum resistance. However, the *lgtB* complemented strain (*lgtB* located in the end of *ompA*) could not recover the serum-resistant phenotype and was even more sensitive than the *lgtB* mutant. In fact, the LOS pattern of the *lgtB* complemented strain was restored to the pattern observed for the wild-type strain. In order to confirm the phenotype of *lgtB* mutant, an in-frame non-polar deletion mutant (Δ*lgtB*::Gm^R^) and an original locus complemented strain were constructed. Results obtained with the non-polar deletion mutant were consistent with those obtained with the previous insertion mutant which indicated that serum sensitive phenotype in *lgtB* mutant may be caused by *lgtB* gene deletion but not by mutation in other genes. The original locus complemented strain showed serum resistance phenotype, indicated that the original locus complementation of the mutation results in a return to the wild type phenotype. Therefore, the serum sensitive phenotype of the previous complemented strain Δ*lgtB*-c may be caused by *lgtB* over-expression.

Host cell invasion is a *H. parasuis* virulence mechanism and the LOS play specific roles in the process (Bouchet et al., 2008; Aragon et al., 2010). The truncated LOS in the Δ*rfaE*, Δ*opsX*, and Δ*rfaF* mutants reduced the adherence and invasion abilities in PUVEC and PK-15 cells (Xu et al., 2013; Zhang et al., 2014). Consistent with previous reports, this study showed impairments in adhesion and invasion of PK-15 cells for both the Δ*lgtB* and Δ*lex-1* mutants, indicating that the *lgtB* and *lex-1* genes were also required for host cell interactions. Compared with the wild-type strain, the Δ*lgtB*-c strain exhibited increased attachment and invasion rates that appeared to be associated with up-regulation of the *lgtB* gene (Figure 6).

## Conclusions

In conclusion, this study investigated the influences of the *lgtB* and *lex-1* genes in the *H. parasuis* SC096 strain on LOS synthesis, serum resistance, adhesion, and invasion. The Δ*lgtB* and Δ*lex-1* mutants caused severe LOS truncations, significant sensitivity to complement-mediated serum and reductions in adherence to and invasion of PK-15 cells. Taken together, the data indicate that the *lgtB* and *lex-1* genes are involved in lipooligosaccharide biosynthesis and may be novel pathogenicity-associated determinants in *H. parasuis*.

## Author contributions

QZ: performed research, analyzed data, wrote paper. SF: analyzed data, wrote paper. JZ: analyzed data. AJ: helped with experiment. KY: helped with experiment. KX: helped with experiment. HF: funded research, analyzed data. ML: funded research, analyzed data.

### Conflict of interest statement

The authors declare that the research was conducted in the absence of any commercial or financial relationships that could be construed as a potential conflict of interest.
