# Supplementary figures and images for: Two Glycosyltransferase Genes of Haemophilus parasuis SC096 Implicated in Lipooligosaccharide Biosynthesis, Serum Resistance, Adherence, and Invasion
Source: Front Cell Infect Microbiol. 2016 Sep 12;6:100. doi: 10.3389/fcimb.2016.00100 (PMC5018477; doi:10.3389/fcimb.2016.00100)

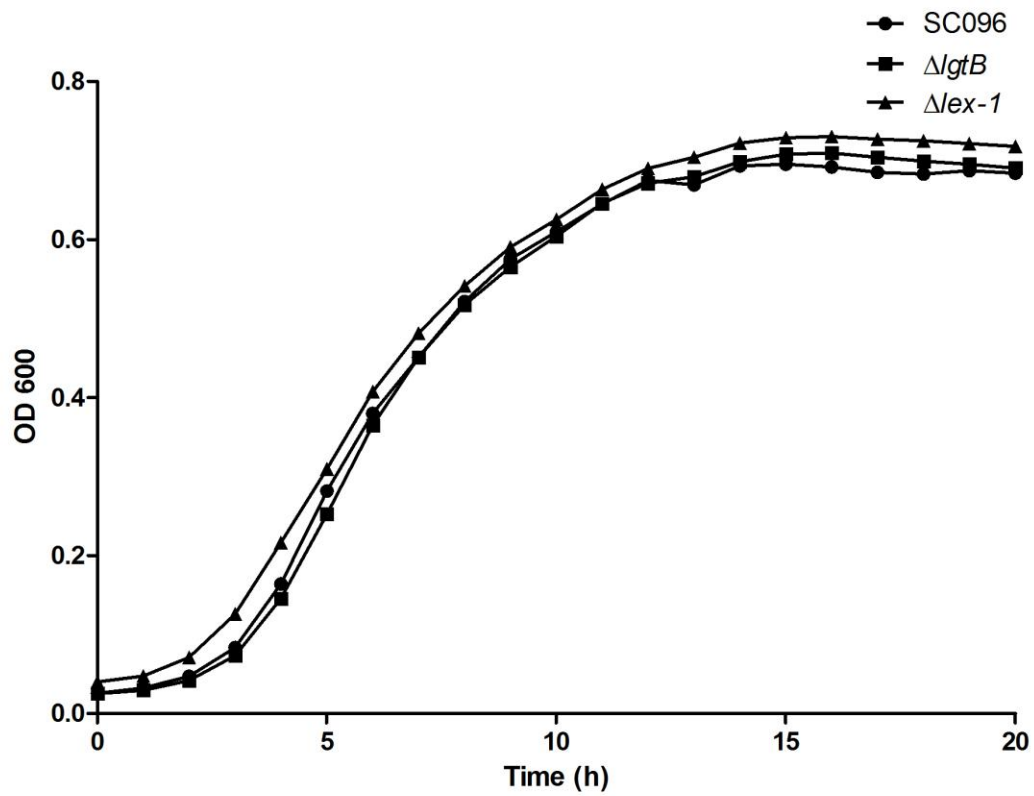

Fig. S1. Growth of the *H. parasuis* SC096, *lgtB* or *lex-1* mutant.

Supplement: Supplementary file 1 [file Image1.PDF]
