# Supplementary material for: Two Glycosyltransferase Genes of Haemophilus parasuis SC096 Implicated in Lipooligosaccharide Biosynthesis, Serum Resistance, Adherence, and Invasion
Source: Front Cell Infect Microbiol. 2016 Sep 12;6:100. doi: 10.3389/fcimb.2016.00100 (PMC5018477; doi:10.3389/fcimb.2016.00100)
Supplement: Supplementary file 2 [file Image2.PDF]

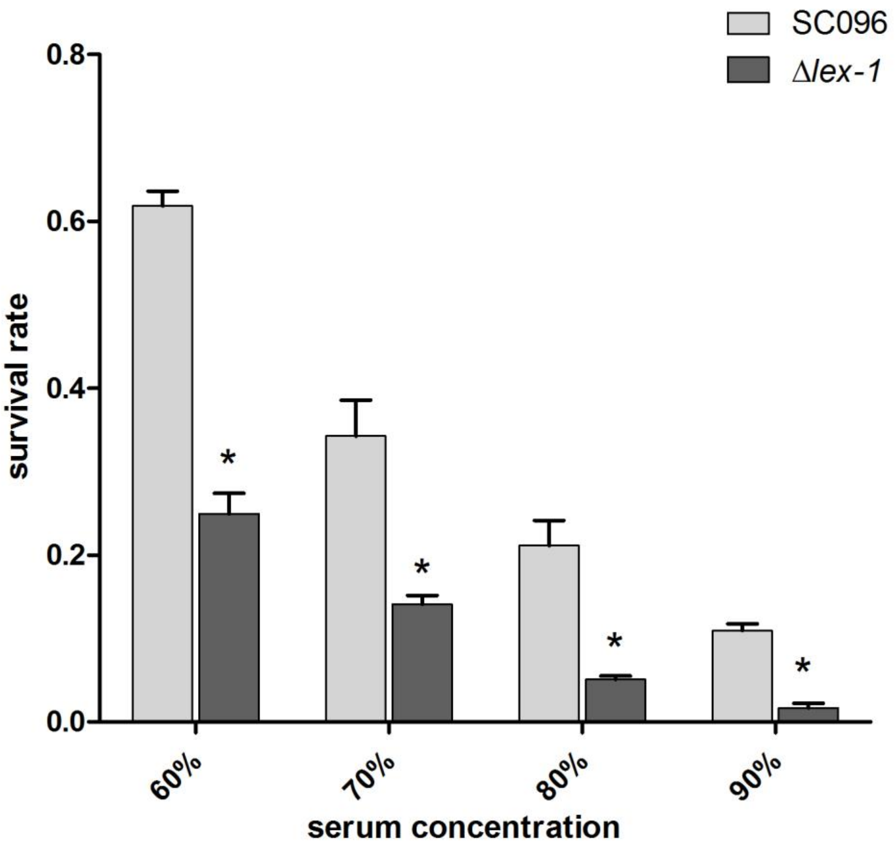


Fig. S2. Survival of *lex-1* mutant treated with porcine serum at different concentrations. The *lex-1* mutant showed significantly increased susceptibility to serum compared with the wild type strain SC096 ($p<0.01$) at different concentrations. Error bars represent the standard deviation of three independent experiments. The asterisks indicate that the survival of bacteria in serum was statistically different ($p<0.01$) from that of the wild-type SC096 strain as judged by the Student *t* test.
